# Role of the BACTEC radiometric method in the evaluation of patients with clinically probable tuberculous meningitis

**DOI:** 10.4103/0972-2327.64637

**Published:** 2010

**Authors:** Jalesh N. Panicker, D. Nagaraja, D. K. Subbakrishna, M. M. Venkataswamy, A. Chandramuki

**Affiliations:** Departments of Neurology, Biostatistics and Neuromicrobiology, National Institute of Mental Health and Neurosciences, Bangalore - 560 029, India

**Keywords:** Early diagnosis, tuberculous meningitis, tuberculoma, cerebrospinal fluid, culture

## Abstract

**Background:**

Confirmation of tuberculous meningitis (TBM) and initiation of treatment are often delayed due to limitations in isolating *Mycobacterium tuberculosis* from cerebrospinal fluid (CSF).

**Objectives:**

To evaluate the role of the BACTEC radiometric method in a clinical setting for the early diagnosis of TBM.

**Materials and Methods:**

Patients meeting criteria for clinically probable TBM over a 3 year period were included. Clinical features, results of CSF investigations (protein, glucose, cell count, Ziehl-Neelsen staining, culture in Löwenstein-Jensen (LJ) medium and BACTEC) and brain CT imaging were reviewed. Drug sensitivity was tested using BACTEC. Patients were started on standard treatment and functional outcome, and response at discharge and follow-up were assessed. Patients were divided according to whether or not *M. tuberculosis* was isolated by BACTEC and the clinical, radiological, and laboratory features compared.

**Results:**

Sixty patients were evaluated. The mean age was 30 years ± 11.7 years. Headache and fever were the most common symptoms and the mean duration was 26 days. CT findings were hydrocephalus (*n*=21), basal exudates (*n*=16), and tuberculoma (*n*=14). In 40 patients, *M. tuberculosis* was isolated by BACTEC and average 15 days was required for detection, whereas it was 30 days in LJ medium. Results of drug-sensitivity testing (*n*=32) were obtained average 7 days after isolation. Patients from whom *M. tuberculosis* had been isolated by BACTEC more often had tuberculomas in CT imaging (*P*=0.018).

**Conclusion:**

Use of the BACTEC method allows early confirmation in patients with clinically probable TBM. It can guide clinicians in the rational use of anti-tuberculosis treatment by confirming diagnosis and identifying drug- sensitivity.

## Introduction

Tuberculosis is a serious public health problem.[1] Though tuberculous meningitis (TBM) accounts for only around 10% of cases, it is the most severe form of extrapulmonary tuberculosis and most common manifestation of central nervous system disease.[2] It is a common cause for chronic meningitis and is associated with significant morbidity and mortality. Early recognition and initiation of treatment are the most important factors predicting favorable outcome in TBM.[2] Though the diagnosis may be suspected based upon clinical features,[3] isolation of *Mycobacterium tuberculosis* from cerebrospinal fluid (CSF) is the gold standard for confirming the diagnosis. The standard tests used include microscopy by Ziehl-Neelsen (ZN) staining, culture in Löwenstein-Jensen (LJ) medium, and polymerase chain reaction. However, limitations in isolating *M. tuberculosis* from CSF using these standard laboratory techniques delay the confirmation of the diagnosis.[2,4,5]

The BACTEC radiometric method has been used for several years for rapid isolation of bacteria.[6] It is based on the measurement of ^14^CO_2_ produced by bacteria when ^14^C labeled palmitic acid present in the liquid media of the culture is metabolized. Recently, the BACTEC method has been used for the isolation of *M. tuberculosis* in CSF specimens. Studies have demonstrated several advantages of using the method, including higher yield and more rapid isolation of *M. tuberculosis*.[7,8] However, studies so far have been laboratory based and we therefore undertook this study to evaluate the role of the BACTEC radiometric method in the clinical setting for patients with clinically probable tuberculous meningitis.

## Materials and Methods

### Clinical evaluation

This study was carried out prospectively over 3 years at a tertiary-care teaching hospital. Patients were diagnosed to have clinically probable TBM if they presented with fever and headache of more than 2 weeks duration and had either (1) abnormal CSF- lymphocytic pleocytosis greater than 20 cells per mm^3^, protein greater than 60 mg/dL, sugar less than 60% of corresponding blood sugar, (2) contrast-enhanced brain computed tomography (CT) study showing basal meningeal enhancement, hydrocephalus, infarcts, or (3) evidence of extraneural tuberculosis.[3] Patients were evaluated by history taking and standard physical and neurological examination. Severity of TBM was staged according to Medical Research Council (MRC) criteria.[9]

### Microbiological Evaluation

#### Clinical specimens

10-15 mL CSF was obtained by lumbar puncture and protein, glucose and cell count were assayed. ZN technique was used to stain for acid-fast bacilli (AFB). Slides were examined under the oil immersion lens for about 30 min, with care taken to view at least 300-500 high power fields and re-examined by an independent examiner.

### CSF Culture

#### BACTEC method

Sterile, uncentrifuged CSF samples were inoculated simultaneously into the BACTEC 12B medium (Becton-Dickinson, USA) and LJ medium (Hi-Media, India).

0.5-1 mL of CSF was inoculated in BACTEC 12B vials, incubated at 37°C, and evaluated for ^14^CO_2_ using the BACTEC 460TB system (Becton Dickinson, USA). As recommended, 0.1 mL of PANTA supplement (polymyxin B 50 µg/mL, amphotericin B 5 µg/mL, nalidixic acid 20 µg/mL, trimethoprim 5 µg/mL and azlocillin 10 µg/mL, Becton Dickinson) was added to each vial prior to inoculation. Culture media were incubated at 37°C for 6 weeks and tested for growth twice weekly for the first 3 weeks and weekly for the subsequent 3 weeks. ^14^CO_2_ production was expressed in terms of the growth Index (GI) and change in GI was proportional to mycobacterial growth. Vials with GI greater than a value of 10 were evaluated daily, and when the GI surpassed 50, specimens were stained with ZN and Gram's stain. BACTEC NAP (*p*-nitro-*α*-acetylamino-*β*-hydroxypropiophenone) test was used to differentiate *M. tuberculosis* from Mycobacteria other than tubercle (MOTT) bacilli.[6] Cultures were negative for growth if there was no significant change in GI after 6 weeks of incubation.

Drug-sensitivity for streptomycin, isoniazid, rifampicin, and ethambutol was tested using the BACTEC system by the modified proportion method.[6]

#### LJ culture

About 0.5 ml of CSF was inoculated on LJ slopes and incubated at 37°C for 8 weeks. The LJ slants were inspected weekly and growth on LJ slants resembling mycobacterial colonies, i.e. rough, tough and buff-colored, were stained with ZN and Gram's stain to confirm the presence of AFB and rule out contamination.

#### Chronic meningitis evaluation

Relevant investigations were performed to exclude other causes for chronic meningitis such as complete blood count, erythrocyte sedimentation rate, infection screen, CSF bacterial and fungal culture, and cytology of cytospin centrifuged samples (Shandon Cytospin, UK).

#### Computed tomography

Computed tomography was performed during the acute stage of illness using third-generation scanner and contrast-enhanced images were reviewed.

#### Treatment and follow up

Patients were started on standard four drug anti-tuberculosis treatment (isoniazid 5-10 mg/kg/day, rifampicin 10 mg/kg/day, pyrazinamide 35 mg/kg/day and ethambutol 20 mg/kg/day or streptomycin 15 mg/kg/day) and prednisolone 1 mg/kg/day, if not contraindicated. Once clinically stabilized, patients were discharged to home and continued on anti-tuberculosis treatment for 3 months and thereafter changed to two-drug regimen with isoniazid and rifampicin. Functional outcome at discharge was graded using the modified Rankin Scale (mRS). This is a seven point scale from 0 (no symptoms) to 6 (death) and patients' outcome was classified as good (mRS≤3) or poor (mRS 4 or 5) according to the evaluation at discharge.[10] Patients were regularly reviewed at the out-patient clinic once at 6 weeks and thereafter every 3-6 months depending upon clinical condition. The functional outcome was reassessed at 6 months.

Patients were divided according to whether or not *M. tuberculosis* was isolated by BACTEC, and clinical, radiological, and laboratory features were compared between the two groups. Statistical analysis was performed using SPSS for Windows version 11.0 (SPSS Inc. Chicago, IL, USA). Chi-square test and Student's t test were used to compare clinical, CSF and radiological findings. *P*-value of <0.05 was considered significant.

## Results

Sixty patients meeting the criteria for clinically probable TBM were evaluated (41 males, 19 females). Clinical features, CSF parameters, and radiological findings are shown in Table 1.

**Table 1:** Clinical, CSF, and radiological features of patients with clinically probable tuberculous meningitis according to whether or not *M. tuberculosis* was isolated by the BACTEC method (*n*=60)

	Total (*n*=60)	BACTEC culture positive (*n*=40)	BACTEC culture negative (*n*=20)	*P* value
Clinical features				
Mean age [mean (range)]	30 (± 11.7 years)	30.4	30	N.S.
Mean duration of fever (days)	26	27.4	25.8	N.S.
Mean duration of headache (days)	18	15.5	23.5	N.S.
Mean duration of impaired consciousness (days)	2.7	2.9	2.4	N.S.
MRC staging (*n*)				
Stage 1	19	11	8	N.S.
Stage 2	6	3	3	N.S.
Stage 3	35	26	9	N.S.
Coexisting extraneural tuberculosis (*n*)	26	15	11	N.S.
HIV infection (*n*)	11	8	3	N.S.
Hyponatremia (*n*)	42	27	15	N.S.
Use of anti-tuberculosis treatment at evaluation (n)	10	4	6	N.S.
CSF findings				
CSF protein, mean (g/L)	1.66	1.55	1.84	N.S.
CSF leukocytes, mean (/µL)	162	134	196	N.S.
CSF glucose, mean (mg/dL)	35	32	39.6	N.S.
CT imaging brain				
Basal exudates (*n*)	16	9	7	N.S.
Hydrocephalus (*n*)	21	14	7	N.S.
Arteritis (*n*)	13	7	6	N.S.
Tuberculoma (*n*)	14	13	1	0.018
Outcome and follow up				
Mortality (*n*)	6	4	2	N.S.
Patients with good outcome at discharge (*n*)*	42	27	15	N.S.
Patients with poor outcome at discharge (*n*)*	12	9	3	N.S.
Follow up (*n*)	49	32	17	
Patients with good outcome at follow up (*n*)*	40	26	14	N.S.
Patients with poor outcome at follow up (*n*)*	9	6	3	N.S.

MRC: Medical Research Council; CSF: cerebrospinal fluid; N.S.: Not significant;

* Patients with scores ≤ 3 on modified Rankin Scale had good outcome and scores 4 or 5 had poor outcome (11)

In 40 patients, *M. tuberculosis* was isolated by BACTEC method and average 15 days was required to detect growth. *M. tuberculosis* was isolated in LJ medium in 15 patients [Table 2] and average 30 days were required to detect growth. Results of drug-sensitivity testing using BACTEC (*n*=32) were obtained within a mean of 7 days (range 5 to 12 days) after isolating *M. tuberculosis*. Three patterns were observed: no resistance to drugs (*n*=20), resistance to isoniazid alone (*n*=11), and resistance to both isoniazid and streptomycin (*n*=1).

**Table 2:** Comparison of isolation of *M. tuberculosis* between BACTEC (*n*=60) and LJ media (*n*=60)

	BACTEC culture positive (*n*=40), n (%)	BACTEC culture negative (*n*=20), n (%)
LJ culture positive (*n*=15)	13 (33)	2 (10)
LJ culture negative (*n*=33)	21 (53)	12 (60)
Contamination in LJ medium (*n*=12)	6 (14)	6 (30)

Differences between the groups were not statistically significant

The mean duration of hospital stay was 13.8 to 14.5 days. Six patients expired. Patients were discharged on anti-tuberculosis treatment and were regularly followed up during out-patient visits. The mean follow-up was 8.9 months. 82% follow-up was obtained and all patients showed favorable response to anti-tuberculosis treatment, without recurrence of meningitis symptoms [Table 1].

Patients in whom *M. tuberculosis* had been isolated from CSF by BACTEC more often had tuberculomas in CT imaging (*P*=0.018) [Table 1]. There were no differences in clinical features, functional outcome, or response to treatment at follow up.

## Discussion

Establishing that the cause for chronic meningitis is due to tuberculosis depends upon microbiological tests. Demonstration of acid-fast bacilli in CSF, either in centrifuged smears or after isolation in culture, is essential for confirming the diagnosis of TBM.[2,5] However, TBM is a paucibacillary condition and the yield from standard investigations is low and results are often delayed. This may be partly due to slow multiplication of *M. tuberculosis* or the strong host immunological response.[2,5,11] The duration of meningitis symptoms as well as CSF parameters such as glucose levels and neutrophil counts may influence bacterial isolation from CSF.[12]

Previous laboratory based-studies that compared isolation of *M. tuberculosis* from CSF samples demonstrated increased sensitivity of the BACTEC radiometric method over culture in LJ medium.[7,8] The present study correlated clinical and radiological features of patients with probable TBM with the results of BACTEC and evaluated the role of this investigation in a clinical setting. The time required for isolating *M. tuberculosis* was less when using the BACTEC method (average 15 days) compared to the standard culture in LJ medium (average 30 days), resulting in earlier confirmation of TBM. Use of the BACTEC method also enabled more rapid assessment of drug-sensitivity, and results were obtained in an average of 7 days after isolation. This is in contrast to the usual 1-2 months required when using LJ medium.[2] Drug resistance was seen in 37% of isolates and isoniazid resistance was most common (*n*=11). Primary drug resistance was more likely, as nine of these patients were never exposed to anti-tuberculosis drugs previously. In ten patients with single drug resistance, second line anti-tuberculosis treatment could not be started due to financial constraints. Incidentally, these patients continued to show good clinical response.

The observation that patients from whom *M. tuberculosis* was isolated in BACTEC more often had tuberculomas requires to be confirmed in a larger series of patients. Necrosis is associated with higher bacillary load and the possibility that some of the lesions had additional necrosis could not be excluded.[13]

The lack of association between isolation in BACTEC and LJ culture may reflect differences between the two media. Several factors are known to contribute to the increased isolation rate in BACTEC including addition of CO_2_ which promotes mycobacterial growth, and antimicrobial agents (PANTA) which reduce contamination.[2,6] However in two patients, *M. tuberculosis* was isolated in LJ medium but not in BACTEC. This may have been due to inherent differences between solid (LJ medium) and liquid media (BACTEC) with regard to the presence of specific growth promoting factors.[7,14] This observation emphasizes the importance of using both types of media when attempting to isolate *M. tuberculosis* from the CSF of a patient with clinically probable TBM.

Comparison between microbiological techniques was beyond the scope of this clinical study. Drawbacks associated with the BACTEC method include involvement of labor-intensive procedures, potential exposure to radio-labeled substrates and expense for set-up and maintenance.[6] These factors would restrict the use of this investigation to tertiary referral centers.

Notwithstanding these drawbacks, our prospective study demonstrates numerous advantages of the BACTEC method that make it a useful investigation for clinicians to confirm the clinical diagnosis of TBM. Patients may have an early confirmation of the diagnosis during the acute stage of the illness. Reports of culture and drug-sensitivity would be available within 3-4 weeks, while a negative report would be available within 6 weeks.
